# Limited use of virtual reality in primary care physiotherapy for patients with chronic pain

**DOI:** 10.1186/s12891-024-07285-5

**Published:** 2024-02-22

**Authors:** Syl Slatman, J. Bart Staal, Harry van Goor, Raymond Ostelo, Remko Soer, Jesper Knoop

**Affiliations:** 1 School of Allied Health, HAN University of Applied Sciences, Nijmegen, the Netherlands; 2 Biomedical Signals and Systems Group, Faculty of Electrical Engineering, Mathematics and Computer Science, University of Twente, Enschede, the Netherlands; 3 Radboud Institute for Health Sciences, IQ Healthcare, Radboud University Medical Centre, Nijmegen, the Netherlands; 4 Department of Surgery, Radboud University Medical Centre, Nijmegen, the Netherlands; 5 Department of Health Sciences, Faculty of Science and Amsterdam Movement Science Research Institute, Vrije Universiteit, Amsterdam, the Netherlands; 6 Department of Epidemiology and Data Science, Amsterdam UMC Location Vrije Universiteit & Amsterdam Movement Sciences, Musculoskeletal Health, Amsterdam, the Netherlands; 7 mProve Hospitals, Zwolle, the Netherlands; 8 Department of Anaesthesiology, Pain Center, University of Groningen, University Medical Center Groningen, Groningen, the Netherlands

**Keywords:** Virtual reality (VR), Chronic pain, Physiotherapy, Survey

## Abstract

**Background:**

Chronic pain is a disabling condition which is prevalent in about 20% of the adult population. Physiotherapy is the most common non-pharmacological treatment option for chronic pain, but often demonstrates unsatisfactory outcomes. Virtual Reality (VR) may offer the opportunity to complement physiotherapy treatment. As VR has only recently been introduced in physiotherapy care, it is unknown to what extent VR is used and how it is valued by physiotherapists. The aim of this study was to analyse physiotherapists’ current usage of, experiences with and physiotherapist characteristics associated with applying therapeutic VR for chronic pain rehabilitation in Dutch primary care physiotherapy.

**Methods:**

This online survey applied two rounds of recruitment: a random sampling round (873 physiotherapists invited, of which 245 (28%) were included) and a purposive sampling round (20 physiotherapists using VR included). Survey results were reported descriptively and physiotherapist characteristics associated with VR use were examined using multivariable logistic regression analysis.

**Results:**

In total, 265 physiotherapists participated in this survey study. Approximately 7% of physiotherapists reported using therapeutic VR for patients with chronic pain. On average, physiotherapists rated their overall experience with therapeutic VR at 7.0 and “whether they would recommend it” at 7.2, both on a 0–10 scale. Most physiotherapists (71%) who use therapeutic VR started using it less than two years ago and use it for a small proportion of their patients with chronic pain. Physiotherapists use therapeutic VR for a variety of conditions, including generalized (55%), neck (45%) and lumbar (37%) chronic pain. Physiotherapists use therapeutic VR mostly to reduce pain (68%), improve coordination (50%) and increase physical mobility (45%). Use of therapeutic VR was associated with a larger physiotherapy practice (OR = 2.38, 95% CI [1.14–4.98]). Unfamiliarity with VR seemed to be the primary reason for not using VR.

**Discussion:**

Therapeutic VR for patients with chronic pain is in its infancy in Dutch primary care physiotherapy practice as only a small minority uses VR. Physiotherapists that use therapeutic VR are modestly positive about the technology, with large heterogeneity between treatment goals, methods of administering VR, proposed working mechanisms and chronic pain conditions to treat.

## Introduction

Approximately one in five adults suffer from chronic pain [1], which mostly occurs in the lower back [2]. Chronic pain is defined as pain lasting longer than three months and is often caused and sustained by a complex interplay of biological, psychological and social factors [3]. Patients with chronic pain report lower quality of life, more social problems, depression and other mental complaints [2, 4] compared to people without chronic pain. Moreover, chronic pain is associated with high direct and indirect societal costs [5].

Treatment options for patients with chronic pain are diverse and include both pharmacological and non-pharmacological possibilities, of which physiotherapy is the most common non-pharmacological treatment [1, 2]. It is recommended to administer stepped care for patients with chronic pain, meaning that treatment modalities of more basic steps (e.g. education, resume normal activities) should be applied before advanced treatment modalities (e.g. physical or psychological therapy) can be considered [6]. During their patient journey, most patients with chronic pain visit a physiotherapist to receive exercises and patient education [1, 2]. However, effects of this treatment are often small to moderate and diminish over time [7, 8], partly due to a lack of treatment adherence of patients [9]. Virtual Reality (VR) could offer a possibility to support physiotherapists in their treatment of patients with chronic pain, amongst other potential mechanisms by motivating patients to keep exercising [10].

VR is an emerging technology in healthcare [11], and is defined as an interactive, 3D computer-generated program in a multimedia environment [12]. VR can be categorized as either immersive or non-immersive, in which immersion usually evokes a greater sense of presence and feeling of being there in the virtual environment (VE). In immersive VR, the user wears equipment, like a head-mounted display (HMD), through which the VE is delivered. In non-immersive VR, the VE is usually delivered through a computer or television screen and controlled using a joystick or other device [13, 14]. Besides motivating patients, proposed working mechanisms of VR for chronic pain include distraction [15], graded exposure therapy [16], relaxation [17] and neurophysiologic alterations [18]. VR has shown to be an effective therapeutic tool in several chronic pain conditions, including fibromyalgia [19, 20], complex regional pain syndrome [21] and chronic low back pain [22, 23]. Besides this, VR in primary care physiotherapy offers possibilities including patient monitoring and at-home treatment, while also offering physiotherapy practices the opportunity to present themselves as innovative [24, 25]. Given the rising healthcare costs, VR could be a useful tool in the treatment of the growing population of patients with chronic pain, by acting as a substitute for treatment or enhancing current treatments as a complementary treatment modality. Moreover, a recent publication by the Dutch Society for Physical Therapy (KNGF) stated that physiotherapy treatment of patients with chronic pain should focus on three core elements: education, self-management and promoting healthy activity behaviour [26]. VR could be of use to aid with each of these goals.

Despite the widespread attention for VR as a treatment tool in chronic pain science and physiotherapy practice, it is not clear to what extent therapeutic VR is being used in primary care physiotherapy in patients with chronic pain. Moreover, it is unclear what the reasons of physiotherapists are to use or not use therapeutic VR, how VR as a treatment modality is being perceived by physiotherapists, and which physiotherapist characteristics are related to VR usage. Results of this study could provide valuable insights for physiotherapists, researchers, policy makers and VR developers to further improve chronic pain treatment of physiotherapists. The aim of this study was to explore physiotherapists’ current usage of, experiences with and characteristics associated with applying therapeutic VR for chronic pain rehabilitation in Dutch primary care physiotherapy.

## Methods

### Design and sample

This cross-sectional, survey study is part of the VARIETY project and funded by ZonMw (project number: 10270032021502), as described in the study protocol [27]. Approval of the ethical research committee of our institution was obtained for this study (HAN ECO: 347.04/22) in compliance with the Declaration of Helsinki. All participants signed online informed consent before participating (tick-box response). The survey data was collected between March and December 2022. This study is reported in line with the Checklist for Reporting Results of Internet E-Survey (CHERRIES) [28].

### Survey

The online open survey was constructed using Google Forms (Appendix 1). Survey questions were based on literature and refined and pilot tested by the research group, by asking four physiotherapists to complete and comment on the online survey before recruitment of participants started. The survey consisted of five demographic questions (i.e. gender, age, practice size, years’ experience as a physiotherapist, physiotherapy specialization), 14 closed-ended questions for physiotherapists that use therapeutic VR (regarding overall experience with therapeutic VR, patients for which therapeutic VR is applied, method of offering therapeutic VR and working mechanisms regarding therapeutic VR) and eight closed-ended questions for physiotherapists that do not use therapeutic VR (regarding reasons not to use therapeutic VR, patients that could use therapeutic VR and possible working mechanisms of therapeutic VR). The order of the questions was constant, without alternation or randomization, adaptive questioning was used to prevent stating redundant questions, reviewing of questions was possible and all questions needed to be answered before completing the survey. The questions were shown on two screens, using a maximum of 13 questions per screen. The survey took physiotherapists approximately five minutes if they use therapeutic VR and two minutes if not.

### Recruitment and sample

**Fig. 1:**
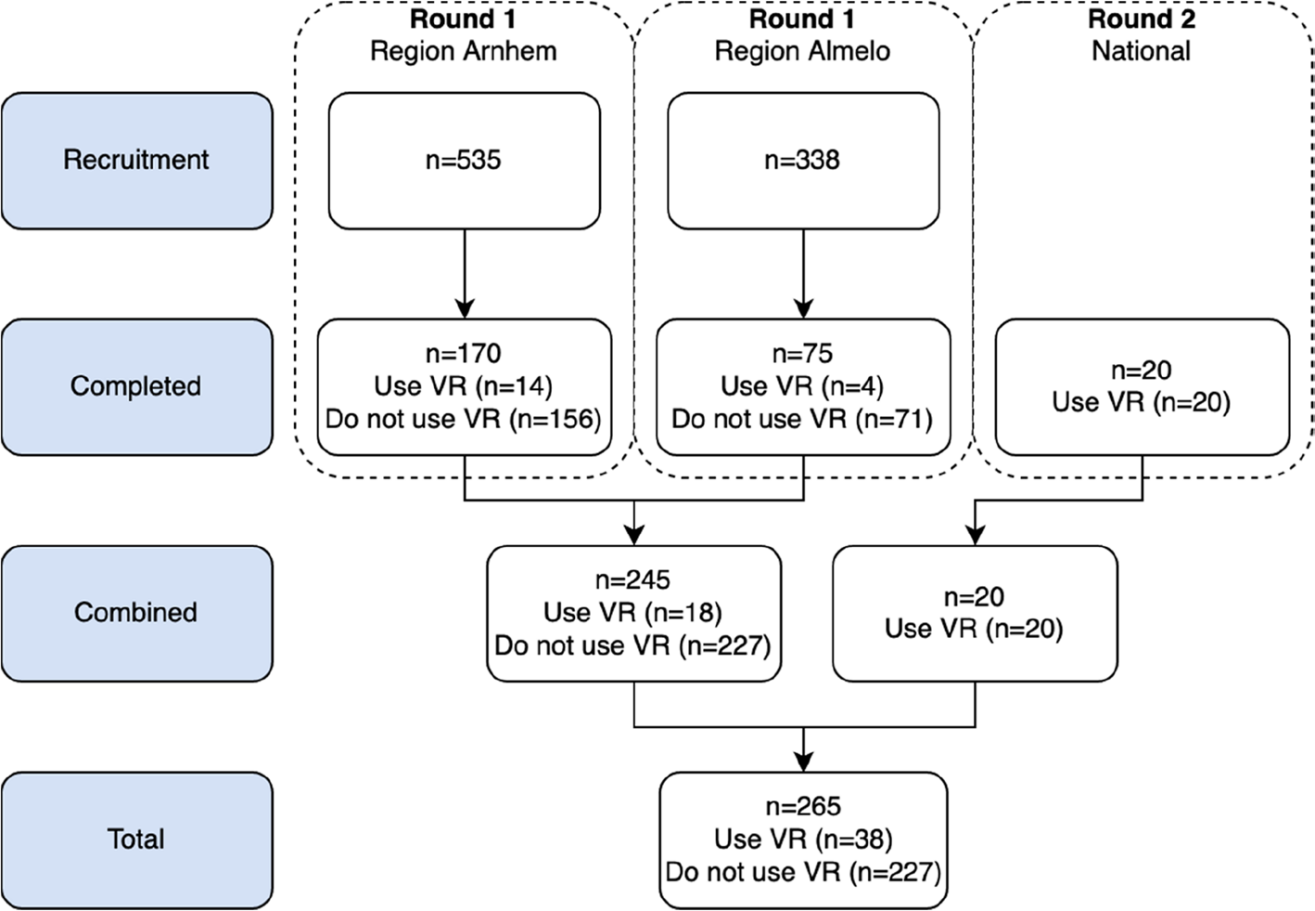
Flow of participating physiotherapists

Data was collected using two consecutive rounds (see Fig. 1). The first round used cluster simple random sampling [29] and included Dutch primary care physiotherapists that did and did not use therapeutic VR for patients with chronic pain. For both rounds, physiotherapists were eligible for participation if they were: (1) practicing primary care physiotherapists that were, (2) working in the Netherlands and, (3) accessible online through e-mail or a contact form. The second round followed a purposive sampling methodology [29], aimed at Dutch primary care physiotherapists who applied therapeutic VR for patients with chronic pain.

#### First round

Participants of the first round of the study were recruited using cluster simple random sampling, in two different regions, namely 40 kms around Arnhem and around Almelo, both cities from the eastern part of the Netherlands. In these two regions, every physiotherapy practice that fulfilled the inclusion criteria was contacted online, regardless if they used therapeutic VR.

#### Second round

Participants in the second round of the study were recruited nationwide using purposive sampling and invited to participate if they provided therapeutic VR for patients with chronic pain.

### Procedure

The initial contact with eligible participants in both rounds consisted of an information letter about the survey and an invitation to voluntarily participate. Two reminders were sent respectively one and two weeks after initial contact to non-responding participants in the first round [30], to reach a minimal retention rate of 25% [31]. All participants voluntarily answered the survey and were not rewarded for participation.

### Analysis

The survey results were downloaded and analysed using SPSS version 27 (IBM corporation, Armonk, NY). Demographic characteristics of physiotherapists were presented using means and standard deviations and frequencies. To examine the association between physiotherapist characteristics (i.e. age, gender, practice size, specialization and years of experience) and therapeutic VR use, a multivariable logistic regression analysis was conducted. For this analysis, the following physiotherapist characteristics were dichotomized: number of years working as a physiotherapist (≤ 9 or ≥ 10 years), specialization (yes/no) and size of physiotherapy practice (≤ 9 or ≥ 10 physiotherapists). The results to the closed-ended questions were analysed by calculating percentages and presented in tables and graphs using GraphPad Prism (GraphPad Inc., San Diego, CA). The surveys from the first round were analysed to gain insight in the prevalence of VR use and characteristics for (non)usage of VR. The survey results of only physiotherapists using therapeutic VR from the first round were combined with the survey results from the second round, to analyse experiences with VR.

## Results

### Inclusion

In total, 873 physiotherapists were invited in the first round. Of these physiotherapists 245 (28%) completed the survey, as shown in Fig. 1. In the second round, 20 physiotherapists that use therapeutic VR completed the survey. All physiotherapists who started the survey completed it.

### VR use in physiotherapy practice

From the total of 245 participating physiotherapists from the first round, 18 (7%) stated that they use therapeutic VR in their treatment of patients with chronic pain, as shown in Table 1. In combination with the 20 physiotherapists using VR from the second round, a total of 38 physiotherapists that use therapeutic VR were surveyed.

**Table 1:** Demographic characteristics of physiotherapists that use and do not use VR

	Round 1		Round 2	Total sample
	Physiotherapists using VR	Physiotherapists not using VR	Physiotherapists using VR	
**N**	18	227	20	265
**Gender, n (%)**				
Female	10 (55.6%)	115 (50.7)	7 (35)	132 (49.8)
Male	8 (44.4)	112 (49.3)	13 (65)	133 (50.2)
**Age, mean (SD)**	37.8 (14.5)	44.4 (13.9)	42.4 (11.4)	43.8 (14)
**Number of years working as a physiotherapist, n (%)**				
0–9 years	7 (38.9)	66 (29.1)	5 (25)	78 (29.4)
9 + years	11 (61.1)	161 (70.9)	15 (75)	187 (70.6)
**Specialization, n (%)**^**a**^				
No specialization	5 (27.8)	65 (28.6)	2 (10)	72 (27.2)
Manual physiotherapy	4 (22.2)	70 (30.8)	13 (65)	87 (32.8)
Sports physiotherapy	1 (5.6)	11 (4.8)	2 (10)	14 (5.3)
Psychosomatic physiotherapy	2 (11.1)	19 (8.4)	4 (20)	25 (9.4)
Paediatric physiotherapy	1 (5.6)	11 (4.8)	0 (0)	12 (4.5)
Pelvic physiotherapy	0 (0)	7 (3.1)	1 (5)	8 (3)
Geriatric physiotherapy	1 (5.6)	12 (5.3)	0 (0)	13 (4.9)
Other	5 (27.8)	53 (23.3)	4 (20)	62 (23.4)
**Size of physiotherapy practice, n (%)**				
1–9 physiotherapists	7 (38.9)	160 (70.5)	11 (55)	178 (67.2)
9 + physiotherapists	11 (61.1)	67 (29.5)	9 (45)	87 (32.8)

^a^more than one option possible

Physiotherapists (*n* = 38) that stated they use VR in their treatment of patients with chronic pain scored their overall experience with therapeutic VR at 7.0 (on a 0 (extremely bad) to 10 (extremely good) scale) and whether they would recommend using therapeutic VR at 7.2 (on a 0 (definitely not) tot 10 (definitely yes) scale). Most physiotherapists (71%) started using therapeutic VR less than two years ago and 82% of physiotherapists use VR for a small proportion (< 10%) of their patients with chronic pain. Physiotherapists use VR at the physiotherapy practice only (39%) or both at practice and patient’s home (61%). Multiple proposed working mechanisms of VR were stated, and educating the patient (58%), relaxation (53%) and activation (53%) were most frequently mentioned. The most commonly reported treatment goals when using VR were pain reduction (68%), coordination improvement (50%) and physical mobility improvement (45%). Regarding VR hardware, nearly everybody (97%) uses HMDs, with a preference for Oculus (56%) and Pico (47%) headsets. Regarding VR software, Reducept (Reducept, Leeuwarden, The Netherlands) is the most used software (50%), followed by Corpus VR (inMotion VR, ‘s-Hertogenbosch, The Netherlands) (26%) and SyncVR (SyncVR, Utrecht, The Netherlands) applications (16%). Physiotherapists using therapeutic VR reported it is mostly used for chronic pain patients between 31–50 years old (90%) and 51–70 years old (82%). The main conditions of patients that receive therapeutic VR are musculoskeletal conditions (53%) and medically unexplained physical symptoms (53%). Within musculoskeletal conditions, VR is mostly applied in patients with generalized pain complaints (55%) and nonspecific cervical (45%) and lumbar (37%) pain (see Table 2).

**Table 2:** Characteristics of usage of therapeutic VR by physiotherapists (*n* = 38)

**Physiotherapists’ experience with therapeutic VR (years), n (%)**	
< 1	10 (26.3)
1–2	17 (44.7)
3–5	10 (26.3)
5 +	1 (2.6)
**Percentage of physiotherapists’ patients that receive therapeutic VR, n (%)**	
0–10%	31 (81.6)
11–25%	5 (13.2)
26–40%	2 (5.3)
**Method of offering therapeutic VR, n (%)**	
Only at home	0 (0)
Only at practice	15 (39.4)
Both	23 (60.5)
**Proposed working mechanisms VR, n (%)**^**a**^	
Education	22 (57.9)
Relaxation	20 (52.6)
Activation	20 (52.6)
Exposure	15 (29.5)
Other	8 (21.1)
**Treatment goal of therapeutic VR, n (%)**^**a**^	
Reduce pain	26 (68.4)
Improve coordination	19 (50)
Improve physical mobility	17 (44.7)
Improve stability	14 (36.8)
Improve strength	9 (23.7)
Improve stamina	9 (23.7)
Other	6 (15.8)
**Therapeutic VR hardware used, n (%)**	
VR headset	37 (97.4)
Nintendo Wii	1 (2.6)
**Type of VR headset used, n (%)**^**a**^	
Pico G2	14 (36.8)
Oculus Go	13 (34.2)
Oculus Quest	9 (23.7)
Pico Neo 3	4 (10.5)
Oculus Rift (S)	2 (5.3)
Samsung Gear	2 (5.3)
HTC Vive	2 (5.3)
Other/unknown	3 (7.9)
**Type of VR software used, n (%)**^**a**^	
Reducept	19 (50)
Corpus VR	10 (26.3)
SyncVR Fit	6 (15.8)
SyncVR Relax & Distract	5 (13.2)
Kana	3 (7.9)
Koji’s Quest	2 (5.3)
Other (commercial) software	3 (7.9)
**Patients’ age for therapeutic VR use, n (%)**^**a**^	
< 18 years	6 (15.8)
18–30 years	28 (73.7)
31–50 years	34 (89.5)
51–70 years	31 (81.6)
70 + years	12 (31.6)
**Patients’ chronic pain conditions for therapeutic VR use, n (%)**^**a**^	
Musculoskeletal conditions	20 (52.6)
Medically unexplained physical symptoms	20 (52.6)
Neurological conditions	7 (18.4)
Geriatric conditions	5 (13.2)
Heart, arterial or lung conditions	2 (5.3)
Oncology	1 (2.6)
Paediatric conditions	0 (0)
Other	6 (15.8)
**Patients’ musculoskeletal conditions for therapeutic VR use, n (%)**^**a**^	
Generalized pain complaints	21 (55.3)
Nonspecific cervical complaints	17 (44.7)
Nonspecific (low) back pain	14 (36.8)
Fibromyalgia	12 (31.6)
Headache or dizziness	10 (26.3)
Arthritis	8 (21.1)
Shoulder or arm complaints	7 (18.4)
Pelvic or hip complaints	2 (5.3)
Other	4 (10.5)

^a^more than one option possible

### Physiotherapist and practice characteristics associated with therapeutic VR use

Results from the multivariable logistic regression analysis among physiotherapists from round one, showed that working at a larger physiotherapy practice (*p* = 0.02) was the only physiotherapy characteristic that was associated with therapeutic VR use, while other physiotherapist characteristics (i.e. age, gender, years working as physiotherapist and specialization) were not found to be associated with therapeutic VR use (see Table 3).

**Table 3:** Physiotherapist characteristics associated with therapeutic VR use

	OR	95% CI	*P*
**Age**	1.02	.99—1.06	.17
**Gender**			
Male	1.00 (reference)		
Female	.70	.34—1.44	.33
**Years working as physiotherapists**			
≤ 9 years	1.00 (reference)		
≥ 10 years	1.24	.46—3.32	.67
**Specialization**			
No	1.00 (reference)		
Yes	2.01	.78—5.19	.17
**Size physiotherapy practice**			
≤ 9 physiotherapists	1.00 (reference)		
≥ 10 physiotherapists	2.38	1.14—4.98	.02*

^*^Statistically significant for *p* < .05; OR = odds ratio

### Physiotherapists that do not use therapeutic VR

From a total of 227 physiotherapists not using therapeutic VR, only 2% has ever used therapeutic VR before and stopped using it, while 98% never used VR. The main reason for not using therapeutic VR is that physiotherapists were unfamiliar with VR as treatment modality (71%), while costs (20%) and lack of eligible patients for VR (18%) are much less reported as reasons (see Table 4).

**Table 4:** Characteristics of physiotherapists that do not use therapeutic VR (*n* = 227)

**Physiotherapists that have ever used therapeutic VR, n (%)**	
Yes, but not anymore	4 (1.8)
No	223 (98.2)
**Reasons for not using therapeutic VR, n (%)**^**a**^	
Costs	46 (20.2)
No suitable patients	40 (17.6)
Unfamiliarity with therapeutic VR	162 (71.4)
Other	28 (12.3)

^a^more than one option possible

## Discussion

This survey among 265 physiotherapists across the Netherlands showed that a minority of approximately 7% of Dutch primary care physiotherapists of the sample population currently uses therapeutic VR in their treatment of patients with chronic pain. Unfamiliarity with VR is the primary reason for not using VR. Larger physiotherapy practices seem to be more likely to use VR compared to smaller practices. Physiotherapists are modestly positive about VR as a treatment modality and use VR for a variety of treatment goals.

The limited VR usage found in our large survey corresponds with that of other therapeutic eHealth technology studies in Dutch physiotherapy care [32, 33]. Ehealth has been introduced in the past two decades amongst other reasons as a strategy to reduce health care costs. Despite the introduction of eHealth, costs of healthcare are still rising in the Netherlands. Studies indicate that many physiotherapists, at least in the Netherlands, are still hesitant to incorporate eHealth in their treatment. For example, one study found that only 1% of patients of physiotherapists in the Netherlands received some form of therapeutic eHealth [34].

In this study, working in a larger physiotherapy practice was associated with using therapeutic VR. This is in line with a previous study that found that eHealth use was associated with physiotherapy practice size [33], possibly due to more financial resources. In contrast to this potential facilitator, implementation of VR in healthcare might be hindered due to technical limitations of the device, lack of comparative research and perceived increased work pressure [35–37]. Some of these barriers were mentioned by the physiotherapists in this survey, but the main reason for surveyed physiotherapists not using therapeutic VR was that they were unfamiliar with using therapeutic VR. This might be related to underexposure of eHealth in physiotherapy programs, as eHealth for example was not mentioned in a recent Delphi study on pain-related content in the Dutch physiotherapy curriculum [38].

Several treatment goals of therapeutic VR were mentioned by physiotherapists, with reducing pain intensity as most commonly reported treatment goal (68%). Therapeutic VR has indeed been found to be effective in reducing acute pain [39], but for chronic pain, the level of evidence for therapeutic VR is weaker and less available [40, 41]. This is surprising since the survey was specifically about treatment of patients with chronic pain. Another treatment goal reported by 50% of the physiotherapists was improving coordination. This can be considered surprising as well, as not improving coordination, but muscle strength or aerobic capacity are more established treatment goals for chronic pain [42], but reported less by the physiotherapists. On the other hand, it could be more difficult to target these established treatment goals by VR.

Another interesting finding in this survey is that therapeutic VR is used less often in older patients (> 70 years old) compared to younger patients with chronic pain. This is in line with previous studies in which healthcare providers tend to not treat older patients with VR, because of existing ageist beliefs that this population for example does not understand VR technology [43]. However, recent studies found that elderly patients with chronic pain could benefit from treatment with VR [44, 45] and find it an acceptable way to manage their pain [46, 47]. This implies there might be possibilities to enhance treatment of older adults with chronic pain by adding therapeutic VR.

One of the strengths of this study was using two rounds of sampling, which made it possible to gain more insight in the reasons for using and not using VR, and in the experiences with VR. Also, by cluster random sampling in two areas in the Netherlands, it was possible to reach an adequate sample size of surveyed physiotherapists. On the other hand, this study had several limitations that should be noted. First, this study focused specifically on primary care physiotherapists and on patients with chronic pain. It is possible that VR use is different in other healthcare settings and for other patient populations. Another limitation is the small sample size of physiotherapists that use therapeutic VR (n = 38), despite efforts to reach this group of physiotherapists using professionals networks and social media. Also, most of these physiotherapists were not very experienced with therapeutic VR for patients with chronic pain. The combination of this sample size with lack of experience with therapeutic VR could impair the generalizability of results about VR usage in clinical practice. Moreover, even though probability sampling (i.e. cluster random sampling) was used to recruit physiotherapists in the first round, it is possible that sampling bias occurred to some extent. For example, because of an increased likelihood of physiotherapists that use VR to respond to the survey rather than physiotherapists that do not use VR. Finally, the survey only included close-ended questions, which might have hindered the collection of more in-depth qualitative information [48]. We chose this to minimize the time for physiotherapists to finish the survey.

Results of this study indicate that therapeutic VR use is still in its infancy in primary care physiotherapy. One possible reason for the low adoption of VR, next to the reported barriers such as costs, is a lack of high-quality evidence on the effectiveness of therapeutic VR for patients with chronic pain [49]. There were some explorative RCTs on therapeutic VR for patients with chronic pain in physiotherapy settings [50–52], but the quality of some of these RCTs is insufficient and limitations to generalize these results include heterogeneity of patient populations and differences between dosage and diversity of used VR software and hardware [53, 54]. Therefore, future research should provide more insights in the (cost-)effectiveness, possible working mechanisms and most suitable patient groups of therapeutic VR for patients with chronic pain, in order to be recommended in clinical guidelines and adopted in clinical practice [49, 55]. Finally, given the novelty and possible increasing usage of therapeutic VR, future research may also focus on replicating the current explorative study after some years to see how therapeutic VR adoption advances in clinical physiotherapy practice [56]. Also, this future research could include open-ended questions to acquire more thorough information and incorporate topics including physiotherapists’ attitudes towards VR, likelihood of VR use and (both physical and mental) symptoms, behaviours and conditions they treat with VR. Finally, for the implementation of VR in physiotherapy care, it would be interesting to gain more insight into values, attitudes and beliefs of physiotherapists that do not use therapeutic VR yet.

Results of this study showed that therapeutic VR for patients with chronic pain is still in its infancy in current Dutch primary care physiotherapy practice, with only 7% of physiotherapists using VR. Unfamiliarity with VR seems to be the primary reason for not using VR. Moreover, larger physiotherapy practices seem to be more likely to use VR compared to smaller practices. This survey also showed that physiotherapists are modestly positive about VR as a treatment modality and that physiotherapists report a large heterogeneity in treatment goals, methods of administering VR, proposed working mechanisms and chronic pain conditions to treat with VR.

## Data Availability

The data generated during this study will not be publicly available, but will be available upon reasonable request to the corresponding author.

## Appendix 1: survey

Questions 1–5: demographic characteristics for all physiotherapists.

Questions 6–19: physiotherapists that use therapeutic VR.

Questions 20–27: physiotherapists that do not use therapeutic VR.

1. What is your gender?
  - Male
  - Female
  - Other
2. What size is the physiotherapy practice you work?
  - 1
  - 2–4
  - 5–9
  - 10–14
  - 15–19
  - 20–24
  - 25–30
  - Over 30
3. How long do you work as a physiotherapists?
  - 0–4 years
  - 5–9 years
  - 10–14 years
  - 15–19 years
  - 20 years or longer
4. What is your age? (open question)
5. Did you specialize as a physiotherapist?*
  - No, I am a regular physiotherapist
  - Yes, as a paediatric physiotherapist
  - Yes, as a manual physiotherapist
  - Yes, as a sports physiotherapist
  - Yes, as a psychosomatic physiotherapist
  - Yes, as a pelvic physiotherapist
  - Yes, other (open question)
6. Did you use therapeutic VR for patients with CMP in the past year?
  - Yes
  - No
7. What is your general experience with therapeutic VR in your physiotherapy treatment? (scale from 0 (very bad) to 10 (very good))
8. To what extent would you recommend therapeutic VR to a colleague physiotherapist? (scale from 0 (would definitely not recommend) to 10 (would definitely recommend))
9. How long have you used therapeutic VR?
  -  < 1 year
  - 1–2 years
  - 3–5 years
  -  > 5 years
10. For what age groups do you use therapeutic VR?*
  -  < 18 year
  - 18–30 years
  - 31–50 years
  - 51–70 years
  -  > 71 years
11. For which conditions do you use therapeutic VR?*
  - Musculoskeletal conditions
  - Medically unexplained physical symptoms
  - Heart, arterial or lung conditions
  - Neurological conditions
  - Geriatric conditions
  - Oncology
  - Paediatric conditions
  - Other
12. For which musculoskeletal conditions do you use therapeutic VR?*
  - Nonspecific cervical complaints
  - Nonspecific (low) back pain
  - Arthritis
  - Headache or dizziness
  - Pelvic or hip complaints
  - Shoulder or arm complaints
  - Fibromyalgia
  - Generalized pain complaints
  - Other
13. For how many patients with CMP do you use therapeutic VR as percentage of your total patient population?
  - 0–10%
  - 11–25%
  - 26–40%
  - 41–60%
  - 61–75%
  - 76–80%
  - Over 80%
14. How do you administer therapeutic VR?
  - Only at practice
  - Only at home
  - Both
15. For which possible working mechanism(s) do you use therapeutic VR?*
  - Education
  - Exposure
  - Relaxation
  - Activation
  - Other
16. For which treatment goals do you use therapeutic VR?*
  - Improve physical mobility
  - Improve stability
  - Improve strength
  - Decrease pain
  - Improve stamina
  - Improve coordination
  - Other
17. Which type of hardware do you use for therapeutic VR?*
  - VR headset
  - Nintendo Wii
  - Xbox Kinect
  - Other
18. Which type of VR headset do you use for therapeutic VR?*
  - Oculus Go
  - Oculus Quest
  - Oculus Rift (S)
  - Samsung Gear
  - HTC Vive
  - Valve Index
  - Vive Force
  - Pico G2
  - Pico Neo 3
  - Other
19. Which type of software do you use for therapeutic VR?*
  - Reducept
  - SyncVR Fit
  - VRelax
  - Corpus VR (InMotion)
  - VRendle
  - Kana
  - SyncVR Relax & Distract
  - Not applicable
  - Other
20. Did you use therapeutic VR in the past?
  - Yes
  - No
21. Why did you stop using therapeutic VR?*
  - Costs
  - Unsatisfied with results
  - Negative experiences patients
  - Negative experiences physiotherapist
  - Other
22. Would you reconsider using therapeutic VR?
  - Yes
  - No
23. Why do you not use therapeutic VR?*
  - Costs
  - I do not treat suitable patients
  - I never informed myself
  - Other
24. For which conditions would you like to use therapeutic VR?*
  - Musculoskeletal conditions
  - Medically unexplained physical symptoms
  - Heart, arterial or lung conditions
  - Neurological conditions
  - Geriatric conditions
  - Oncology
  - Paediatric conditions
  - Other
25. For which possible working mechanism(s) would you like to use therapeutic VR?*
  - Education
  - Exposure
  - Relaxation
  - Activation
  - Other
26. For which treatment goals would you like to use therapeutic VR?*
  - Improve physical mobility
  - Improve stability
  - Improve strength
  - Decrease pain
  - Improve stamina
  - Improve coordination
  - Other
27. Why do you not use therapeutic VR?*
  - Costs
  - I do not treat suitable patients
  - I never informed myself

*more than one option possible.

## Abbreviations

HMD: Head-mounted display
KNGF: Dutch Society for Physical Therapy
VE: Virtual environment
VR: Virtual reality
